# 

**Published:** 1918-11

**Authors:** 


### CONTENTS FOR THE NOVEMBER ISSUE.

EDITORIALS.

War Opportunities.................................... 147
Chemistry and the War................................ 150
Meeting of Inter-Allied Surgeons..................... 151
Former Epidemics..................................... 156
Diphtheria Anti-Toxin Specific for Influenza......... 157
Report of the Meeting of The Medical Association of the Southwest ................................... 158
M. M. Smith, M. D., Dallas, Texas.................... 161

ORIGINAL ARTICLES.

Gout. By N. D. Buie, M. D., Marlin, Texas............ 163
Prevention of Diseases in the War.................... 168

News Items........................................... 174

Army News............................................ 176

Personals............................................ 179

Deaths............................................... 180

Jokes................................................ 181

